# Effects of container type and size on thermal processing characteristics and B-vitamin retention of canned cat food

**DOI:** 10.3389/fvets.2023.1175819

**Published:** 2023-05-11

**Authors:** Amanda N. Dainton, Lydia M. Molnar, Charles Gregory Aldrich

**Affiliations:** Department of Grain Science and Industry, Kansas State University, Manhattan, KS, United States

**Keywords:** B-vitamins, canned cat food, packaging, retention, riboflavin, thermal processing, thiamin, wet cat food

## Abstract

**Introduction:**

Rigid cans were the traditional container for canned cat foods, but semi-rigid trays/tubs and flexible pouches are popular options as well. Despite this, little is published on the effects of canned cat food container characteristics on thermal processing and retention of B-vitamins. Therefore, the objective was to evaluate the effects of container size and type on thermal processing and B-vitamin retention.

**Materials and methods:**

Treatments were arranged in a factorial with two container sizes [small (85–99 g) and medium (156–198 g)] and three container types (flexible, semi-rigid, and rigid). A canned cat food formula was prepared, filled, and sealed into containers before retort processing to a heating cycle target lethality of 8 min. Internal retort and container temperatures were used to calculate accumulated lethality. Thiamin, riboflavin, niacin, pantothenic acid, pyridoxine, biotin, folic acid, cobalamin, and moisture contents were analyzed in pre- and post-retort samples by commercial laboratories. Thermal processing metrics were analyzed (SAS v. 9.4; SAS Institute, Cary, NC) with the fixed effects of container size, container type, and their interaction. Dry matter basis B-vitamin contents were analyzed with container size, container type, processing stage, and all two-way and three-way interactions as fixed effects. Means were separated using Fisher's LSD at a *P*-value < 0.05.

**Results and discussion:**

Total accumulated lethality was greater (*P* < 0.05) for semi-rigid and flexible containers (average 14.99 min) than for rigid containers (12.86 min). The greater processing of semi-rigid and flexible containers was likely influenced by required retort settings. Thiamin and riboflavin contents decreased (*P* < 0.05) by 30.4 and 18.3%, respectively, due to retort processing. Niacin, biotin, and cobalamin were not affected (*P* > 0.05) by processing. Processing increased (*P* < 0.05) pantothenic acid (9.1%), pyridoxine (22.6%), and folic acid (22.6%). This was likely caused by sampling or analytical variation. No interaction involving processing stage was significant for any B-vitamin (*P* > 0.05). B-vitamin retention was not influenced by differences in thermal processing caused by the packaging treatments. Thiamin and riboflavin were the only B-vitamins meaningfully impacted by processing and retention was not improved by any container characteristic.

## 1. Introduction

Many pet cats are fed commercial foods designed to meet their nutritional requirements. These foods come in many formats, including canned foods. The term “canned” is defined as “… processed, commercially sterilized, and sealed according to 21 CFR part 113 in hermetically sealed containers such as but not limited to cans, pouches, tubs, and trays” (1). The Code of Federal Regulations (CFR) defines commercially sterilized as it applies to pet food as the absence of microorganisms and spores that pose a public health concern and microorganisms that reproduce at room temperature after a heat process (2). A retort, or a commercially-sized pressure cooker, is used to achieve commercial sterility of food products. The duration of time the retort is held at specific temperatures to achieve commercial sterility is termed the scheduled process (2). Foods processed with a retort must be contained within hermetically sealed containers, which are sealed in a way that prevents matter from moving into or out of the container (3).

As described in the Association of American Feed Control Officials (AAFCO) definition, canned pet food can be processed in different types of containers. The United States Department of Agriculture Food Safety and Inspection Service (USDA-FSIS) categorizes containers for thermal processing into three groups: flexible, semi-rigid, and rigid. These categories are defined by whether the food product significantly influences container shape and 0.7 kg/cm^3^ of force under normal atmospheric conditions can change the container shape (3). As such, the internal food product only influences the shape of flexible containers and semi-rigid containers can deform with <0.7 kg/cm^3^ of force, while rigid containers require forces >0.7 kg/cm^3^ for deformation. While the rigid metal can is the most common container type, other options for canned cat foods include flexible pouches and semi-rigid trays (4). The popularity of these containers can be attributed to improved consumer convenience (3), but few peer-reviewed publications regarding canned cat food assess these container types.

Thiamin (vitamin B1) is not stable during retort processing and has been a focus in canned pet food research. Consumption of a diet deficient in thiamin by cats can cause severe neurological deficits such as decreased ability to learn in kittens (5) and seizures and ventroflexion in cats of all ages (6–8). This deficiency can become deadly quickly with reports of death within 2 or 3 days once movement is visibly difficult if treatment is not provided (9). It is the responsibility of pet food companies to ensure their products contain enough thiamin before they enter the marketplace. However, thiamin-deficient canned cat foods are still found on store shelves (10) even when pet food companies do their best to prevent this. At the same time, other B-vitamins (riboflavin, niacin, pantothenic acid, pyridoxine, folic acid, biotin, and cobalamin) also have recommended levels for inclusion in canned cat foods (1). Many of the B-vitamins interact with each other and deficiency of a single vitamin can have far-reaching consequences. For example, pyridoxine and folic acid are needed for ideal absorption of cobalamin and thiamin, respectively (11). Identification of a B-vitamin deficiency is challenging and often deficiencies of individual B-vitamins present similarly. As such, pet food companies need to ensure these vitamins also survive thermal processing.

The objective of this experiment was to determine the effects of packaging type and size on thermal processing characteristics and B-vitamin retention. The first hypothesis was that smaller containers made of flexible materials would achieve the target lethality faster than medium containers made of rigid materials. However, the second hypothesis was that retention of heat-labile B-vitamins would not be impacted by container type and size when processed to similar lethalities.

## 2. Materials and methods

### 2.1. Packaging types and sizes

Container types and sizes were chosen to encompass all categories of packaging materials and the container sizes most often used for commercial canned cat foods for a total of six containers. Container types were categorized as flexible, semi-rigid, and rigid based on USDA-FSIS guidelines (3) and container sizes were categorized as small and medium based on target fill weights. The small (85 g target fill weight; 95 mm width × 138 mm height × 25 mm gusset) and medium (170 g target fill weight; 140 mm wide × 180 mm high × 25 mm gusset) flexible containers used were made of multiple layers. Specifically, the small flexible container consisted of 12 μ PET, adhesive, Al Foil 8 μ, adhesive, and polypropylene 70 μ and the medium flexible container consisted of 12 μ PET, adhesive, Al Foil 7 μ, adhesive, Nylon 15 μ, adhesive, and polypropylene 60 μ. Small (99 g target fill weight) and medium (198 g target fill weight) semi-rigid containers were made of layers of polypropylene (1.2 mm for small and 1.4 mm for medium), EVOH 10%, and a polypropylene base sheet. Rigid containers were two-piece aluminum cans with modified vinyl coatings and target fill weights of 85 g (small; 209.5 × 107) and 156 g (medium; 307 × 109.3).

### 2.2. Canned cat food batter production

The canned cat food formula was designed to mimic a generic chicken-based commercial canned cat food (Table 1). This formula was intended to contain 78.0% moisture, 35.0% crude protein on a dry matter basis (DMB), 44.3% crude fat DMB, and 8.0% ash DMB with a pH of 6.5. Successful formulation was verified with analysis of pre- and post-retort moisture and pH and post-retort crude protein, crude fat, and ash (Supplementary Table 1). Vitamin premix level was ~10× the typical production level to ensure high enough concentrations of B-vitamins for chemical analysis. The target levels of B-vitamins before retort processing on a DMB were 3,209.0 mg/kg thiamin, 90.9 mg/kg riboflavin, 909.1 mg/kg niacin, 136.4 mg/kg pantothenic acid, 272.7 mg/kg pyridoxine, 1.60 mg/kg biotin, 22.2 mg/kg folic acid, and 0.60 mg/kg cobalamin.

**Table 1:** Canned cat food formula super-fortified with B-vitamins.

**Ingredient**	**Percentage, %**
Water and steam	39.149
Mechanically separated chicken	55.333
Brown rice	3.000
Dried egg product	0.500
Potassium chloride	0.500
Soybean oil	0.500
Guar gum	0.350
Mineral premix	0.250
Vitamin premix^a^	0.200
Choline chloride	0.092
Kappa carrageenan	0.050
Taurine	0.041
Salt	0.035

a Target B-vitamin content in processed cat foods was 3,209.0 mg thiamin, 90.9 mg riboflavin, 909.1 mg niacin, 136.4 mg pantothenic acid, 272.7 mg pyridoxine, 1.60 mg biotin, 22.2 mg folic acid, and 0.60 mg cobalamin per kg of diet dry matter.

An independent batch of pre-retort batter (69 kg) was made for each replicate (*n* = 2) of each combination of package type (*n* = 3) and size (*n* = 2) for a total of 12 independent diet productions. Frozen blocks of mechanically separated chicken (Protein Inc/BHJ, St Joseph, MO) were ground through a pilot plant extructor and grinder with 9.5 mm openings and weighed into a horizontal jacketed mixer (Rietz, Evansville, IN). Brown rice (Gulf Pacific Rice, Houston, TX), dried egg product (Rose Acre Farms, Seymour, IN), potassium chloride (Prince Agri Products Inc., Quincy, IL), soybean oil (preserved with mixed tocopherols; Columbus Foods, Des Plaines, IL), guar gum (Intercolloid, Wembley, Middlesex, UK), mineral premix (Prince Agri Products Inc., Quincy, IL), choline chloride (SEM Minerals, Quincy, IL), kappa carrageenan (Marcel Trading, Quezon City, Philippines), taurine (Prinova, Carol Stream, IL), and salt (Cargill, Hutchison, KS) were added to the mixer and blended for 5 min until uniform. Directly injected steam was used to bring the batter up to 43.3°C and water was added to bring the moisture content up to 78% (verified by rapid methods; CEM, Mathews, NC). At this time, a sample of the raw batter was taken for chemical analyses to capture the background vitamin content provided by the non-vitamin premix ingredients in the formula.

Next, the vitamin premix (DSM Nutritional Products, LLC, Parsippany, NJ) was added to the batter and mixed for 10 min prior to homogenization with 3 and 6 mm die plates (Karl Schnell Emulsifier, Winterbach, Germany). A sample was taken at this processing stage to capture the pre-retort moisture and B-vitamin contents.

### 2.3. Thermal processing of canned cat foods

Each replicate of each combination of packaging type and packaging size was thermally processed separately. Prior to container filling, a select number of packages (16 for small and medium rigid cans and 14 for small and medium flexible pouches and semi-rigid trays/tubs) were outfitted with type-T thermocouples (Ecklund-Harrison Technologies Inc., Ft. Meyers, FL). This number was chosen to allow for extra thermocouples beyond the minimum ten recommended by the Institute for Thermal Processing Specialists (12) in the event of thermocouple failure during retort processing. Additionally, two thermocouple leads were used to monitor temperature inside the retort during processing.

Fifty containers, including those with thermocouples, were filled with their respective target weight of batter and hermetically sealed with a sealer (small and medium cans: Ferrum, Schafesheim, Switzerland; small trays: Shinwa, Shinwa, Japan; medium trays: Raque Food Systems, Louisville, KP; small and medium pouches: PMP mini vacuum seamer, PakSource Global LLC., Sarasota, FL). Satisfactory closer was confirmed with pre- and post-retort processing burst tests for the flexible and semi-rigid containers and with post-retort vacuum measurements for the rigid containers (Supplementary Table 1).

**Figure 1:**
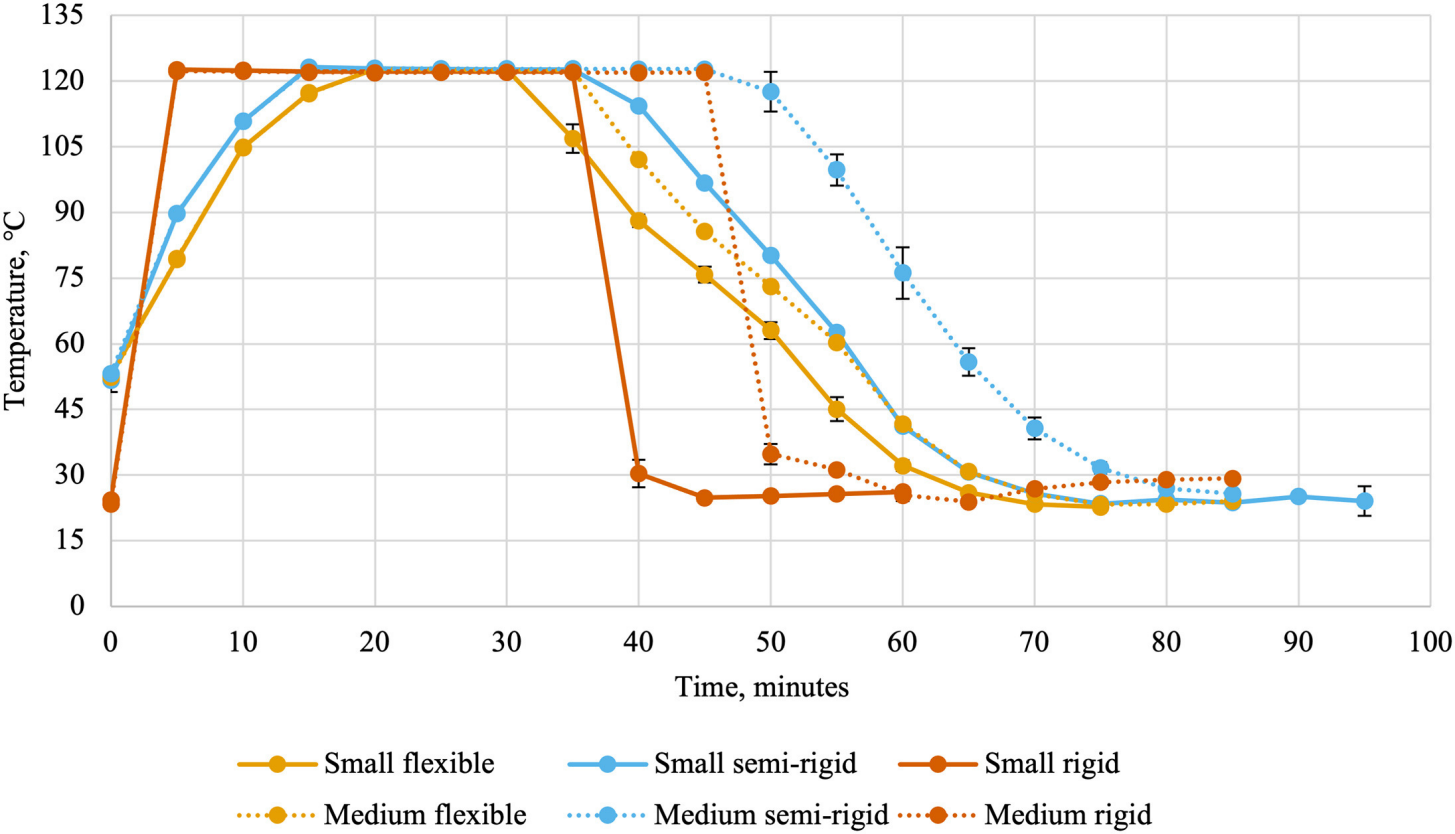
Retort temperatures (average ± standard deviation) every 5 min during thermal processing of canned cat food processed in containers of two different sizes and three different types.

**Figure 2:**
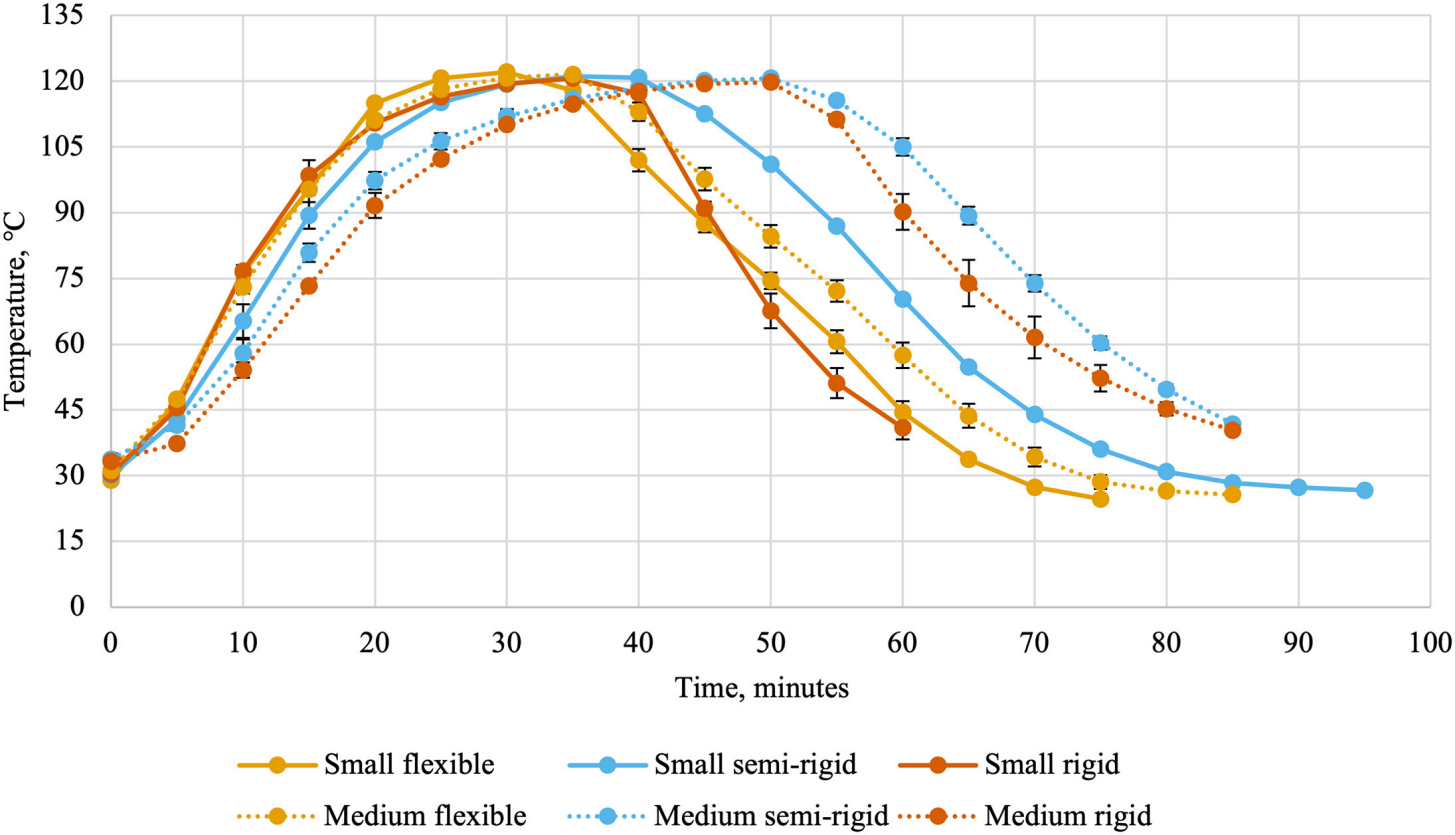
Internal can temperatures (average ± standard deviation) every 5 min during thermal processing of canned cat food processed in containers of two different sizes and three different types.

Ballast containers comprised the two layers of containers above and below the experimental treatments to mimic a commercial production. Thermocouples and leads were attached to the data collection system (CALSoft v. 5.0.5.; TechniCAL LLC, Metairie, LA) at the start of retort processing. Separate retorts were used for the different packaging types due to their behavior during retort processing (cans: Reid Boiler Works Inc. Bellingham, WA; trays and pouches: JBT, Madera, CA). Temperatures inside the retort (Figure 1) and inside containers (Figure 2) were recorded every 30 s and used to calculate lethality (Equation 1) by the data collection system using the trapezoidal method (Equation 2). In these equations, T_C_(t) represented the temperature recorded by a container's thermocouple at time t and Δt represented the time interval between measurements in minutes (0.5). The retort automatically entered the water cooling phase once the last thermocouple reached the minimum lethality value of 8 min. Temperatures were recorded during the cooling cycle to allow for calculation of accumulated lethality during the heating cycle, cooling cycle, and throughout the entire scheduled process. The units of lethality are equivalent minutes of processing at 121.11°C. As such, greater lethality values indicate more intense processing.


(1) $$\begin{array}{l} Lethality=\int 10^{\frac{T_{C}\left(t\right)-121.11{}^{\circ}C}{10{}^{\circ}C}}\Delta t \end{array}$$

(2) $$\begin{array}{l} Lethality=\overset{t}{\underset{0}{\sum }}10^{\frac{T_{C}\left(t\right)-121.11{}^{\circ}C}{10}}\Delta t \end{array}$$

Once cooled, packages were removed from the retort and stored at ambient temperature overnight. The following day, four containers from each replicate were combined to create a composite sample of the post-retort diet for chemical analysis.

### 2.4. Chemical analyses

Pre-retort batters (before and after the addition of vitamin premix) and post-retort diets were analyzed in duplicate for moisture (AOAC 934.01), and thiamin [EN 14122:2003; (13)] at a commercial laboratory (Nestle Purina Analytical Laboratory, St. Louis, MO). Riboflavin (AOAC 944.33), niacin (AOAC 944.13), and pyridoxine (AOAC 961.15) contents were measured in duplicate in pre-retort batters and post-retort diets by the vitamin premix supplier (DSM Nutritional Products, LLC; Parsippany, NJ). Pre-retort batters and post-retort diets were analyzed once for pantothenic acid (AOAC 945.74), biotin (14), folic acid (AOAC 992.05), and cobalamin (AOAC 952.20) by a commercial laboratory (Covance Laboratory, Madison, WI). Analysis of crude protein (AOAC 990.03), crude fat (AOAC 920.39), and ash (AOAC 942.05) in post- retort diets was conducted in duplicate by a commercial laboratory (Nestle Purina Analytical Laboratory, St. Louis, MO). pH readings were taken of pre-retort batters during production and of post-retort diets after diet production and sampling.

### 2.5. Statistical analysis

Moisture content, crude protein content, crude fat content, ash content, and pH were presented as average values ± standard deviation. Initial can temperature, time to reach target lethality and accumulated lethality values were analyzed as a 2 × 3 factorial with the fixed effects of container type and container size (GLIMMIX procedure, SAS v. 9.4; SAS Institute, Cary, NC). B-vitamin contents were analyzed as a 2 × 3 × 2 factorial with the main effects of container size (small and medium), container type (flexible, semi-rigid, and rigid), and processing stage (pre- and post-retort processing), and all two-way and three-way interactions. An analysis of variance was employed to determine the significance of the models' main effects and interactions. Fisher's least significant difference was used to separate means at an α of 0.05.

## 3. Results

### 3.1. Thermal processing metrics

Thermocouple failure occurred during the processing of small flexible pouch replicates 1 and 2 (four and two failures, respectively), small semi-rigid tray/tub replicates 1 and 2 (two failures for both), small rigid can replicate 1 (one failure), medium flexible pouch replicates 1 and 2 (two and three failures, respectively), and medium rigid can replicate 1 (one failure). No thermocouple failures occurred during processing of medium semi-rigid trays/tubs.

On average, medium size containers were initially warmer (*P* < 0.05) than small size containers (32.21 vs. 29.73°C; Table 2). However, there was no difference (*P* > 0.05) in initial container temperatures between container types or the interaction of container size and type. Medium size containers (41.6 min) required 23.4% more time to achieve the target lethality of 8 min than small size containers (33.7 min). The amount of time required for rigid and semi-rigid containers (40.9 min) was similar (*P* > 0.05) but greater than for flexible containers (31.2 min). These relationships were observed for the interaction of container size and type, wherein medium rigid and semi-rigid containers (average 42.0 min) needed 17.3% more time (*P* < 0.05) than small rigid and semi-rigid containers (average 35.8 min). Medium flexible containers (32.8 min) required less time (*P* < 0.05) and small flexible containers (29.5 min) required the least (*P* < 0.05) amount of time overall.

**Table 2:** Thermal processing metrics for a canned cat food processed in two different container sizes and three different container types.

**Measurement**	**Small**			**Medium**			**Size × Type SEM^*a*^**	***P*** **-values**		
	**Flexible**	**Semi-rigid**	**Rigid**	**Flexible**	**Semi-rigid**	**Rigid**		**Size**	**Type**	**Size** × **type**
Initial can temperature, °C	28.91	30.02	30.26	31.15	33.77	32.01	0.874	0.0111	0.1809	0.5292
Time to target lethality, min	29.5	36.1	35.5	32.8	46.4	45.6	0.63	<0.0001	<0.0001	0.0023
Total lethality, min	15.02	15.14	12.67	14.43	15.35	13.05	0.510	0.9939	0.0077	0.6178
Heating lethality, min	10.23	9.28	9.23	10.42	9.49	9.76	0.435	0.4160	0.1365	0.9096
Cooling lethality, min	4.79	5.87	3.45	4.01	5.88	3.06	0.150	0.0194	<0.0001	0.0970

a SEM, standard error of the mean.

Lethality accumulated during the heating cycle was not affected (*P* > 0.05) by container size, type, or their interaction (average 9.74 min; Table 2). However, lethality during the cooling cycle was 9.0% greater (*P* < 0.05) for small containers than for medium containers (4.70 vs. 4.31 min). Semi-rigid containers (5.87 min) accumulated more (*P* < 0.05) lethality during the cooling cycle than rigid containers (3.25 min) with cooling cycle lethality for flexible containers (4.40 min) intermediate. Differences in the total lethality accumulated during retort processing were observed between container types, with flexible and semi-rigid containers (average 14.99 min) accumulating 16.6% more (*P* < 0.05) lethality than rigid containers (12.86 min).

### 3.2. B-vitamin content of pre- and post-retort samples

Riboflavin, niacin, and pyridoxine levels in pre-retort samples before the addition of vitamin premix fell below detectable limits. Pantothenic acid (21.8 mg/kg DMB), biotin (0.319 mg/kg DMB), folic acid (0.509 mg/kg DMB), and cobalamin (0.0588 mg/kg DMB) were lower prior to inclusion of the vitamin premix. Thiamin was only detectable in one sample of pre-retort batter without the vitamin premix, resulting in an average thiamin content of 2.57 mg/kg DMB for one replicate of medium pouches prior to vitamin premix addition.

**Figure 3:**
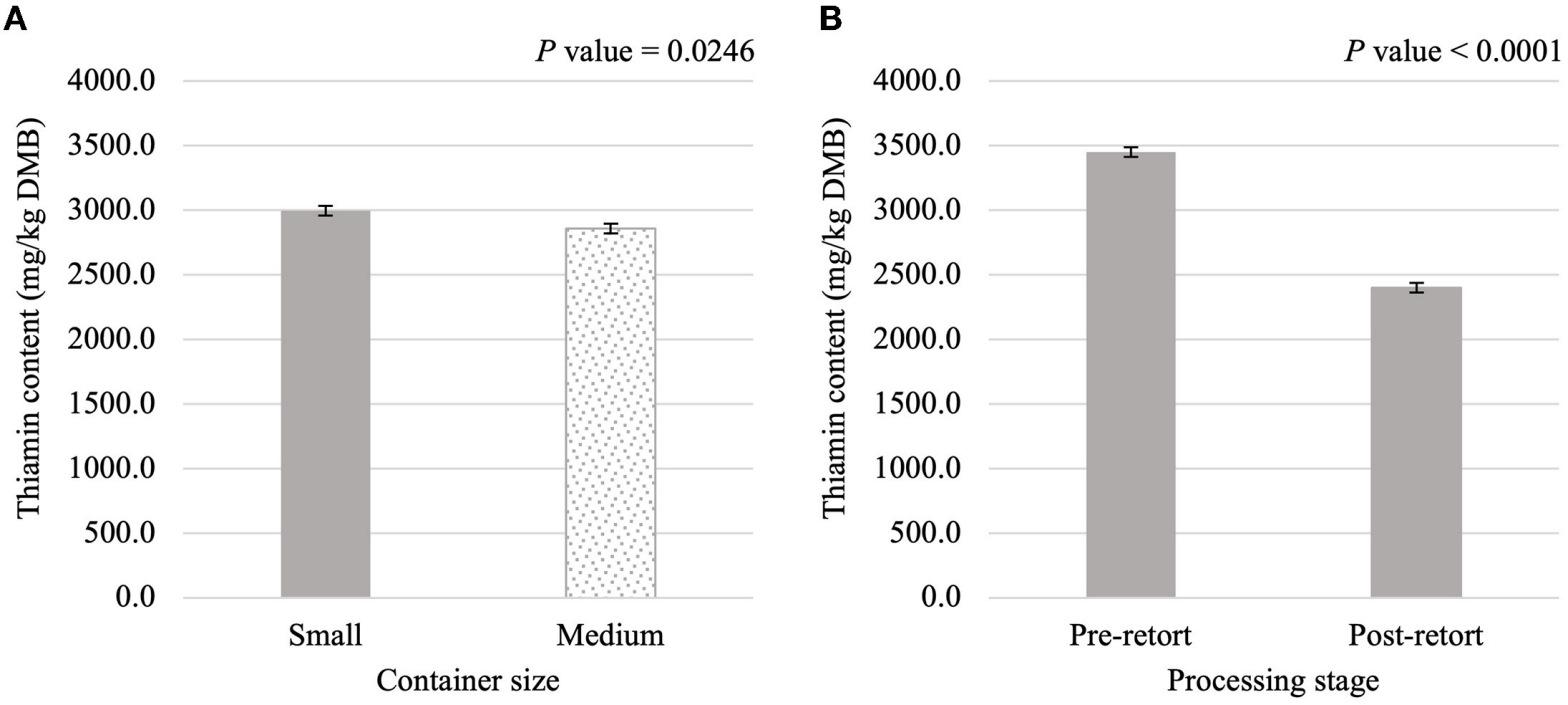
Main effects of container size **(A)** and processing stage **(B)** on dry matter basis (DMB) thiamin content (average ± pooled standard error of the mean) of canned cat food processed in containers of two different sizes and three different types.

**Figure 4:**
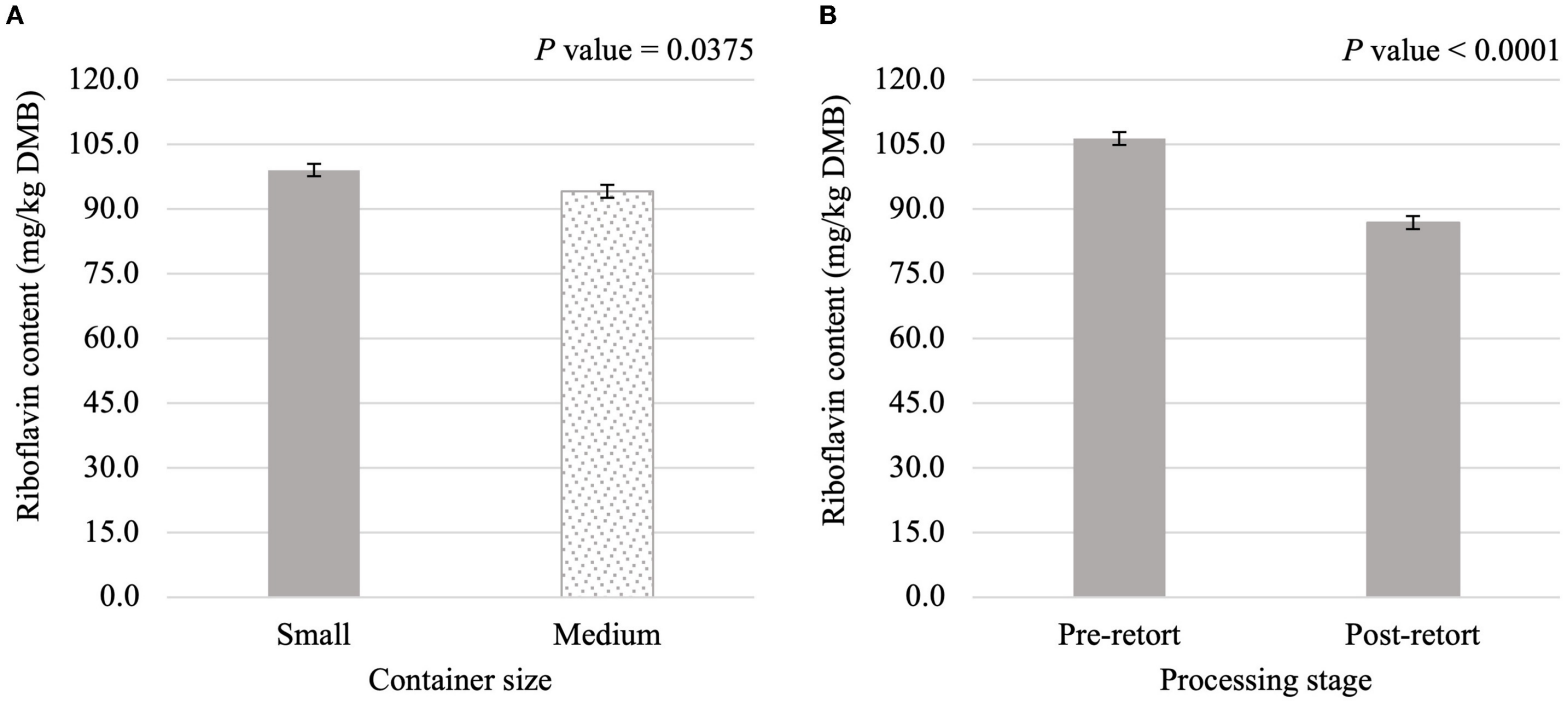
Main effects of container size **(A)** and processing stage **(B)** on dry matter basis (DMB) riboflavin content (average ± pooled standard error of the mean) of canned cat food processed in containers of two different sizes and three different types.

Thiamin content was affected (*P* < 0.05) by the main effects of container size and processing stage. On average, small containers (2,996.1 mg/kg DMB) contained 4.8% more thiamin than medium containers (2,858.0 mg/kg DMB) separate from container size and/or processing stage (Figure 3A). Not considering container size or type, thiamin content decreased by 30.4% due to retort processing (Figure 3B). However, the main effect of container type and all interactions were not significant (*P* > 0.05; Table 3). Similar relationships were observed for riboflavin content, where small containers contained 5.2% more (*P* < 0.05; Figure 4A) riboflavin than medium containers and degradation due to retort processing was 18.3% (*P* < 0.05; Figure 4B).

**Table 3:** *P*-values for a factorial arrangement of treatments evaluating the effects of two container sizes, three container types, and two processing stages on the content of B-vitamins in a canned cat food.

**Vitamin**	**Size**	**Type**	**Processing stage**	**Size × type**	**Size × processing stage**	**Type × processing stage**	**Size × type × processing stage**
Thiamin	0.0246	0.6825	<0.0001	0.1824	0.4238	0.2173	0.8322
Riboflavin	0.0375	0.3506	<0.0001	0.6230	0.2428	0.3796	0.7100
Niacin	0.0339	0.5214	0.0891	0.6519	0.9151	0.1252	0.7032
Pantothenic acid	0.0529	0.2256	0.0012	0.6115	0.8647	0.6115	0.0927
Pyridoxine	0.5003	0.0686	0.0009	0.2299	0.2442	0.7504	0.7747
Biotin	0.4984	0.4316	0.9533	0.4504	0.5045	0.1137	0.2669
Folic acid	0.2368	0.0208	<0.0001	0.4251	0.0320	0.3061	0.6058
Cobalamin	0.5797	0.2461	0.3723	0.1850	0.9521	0.8663	0.8208

**Figure 5:**
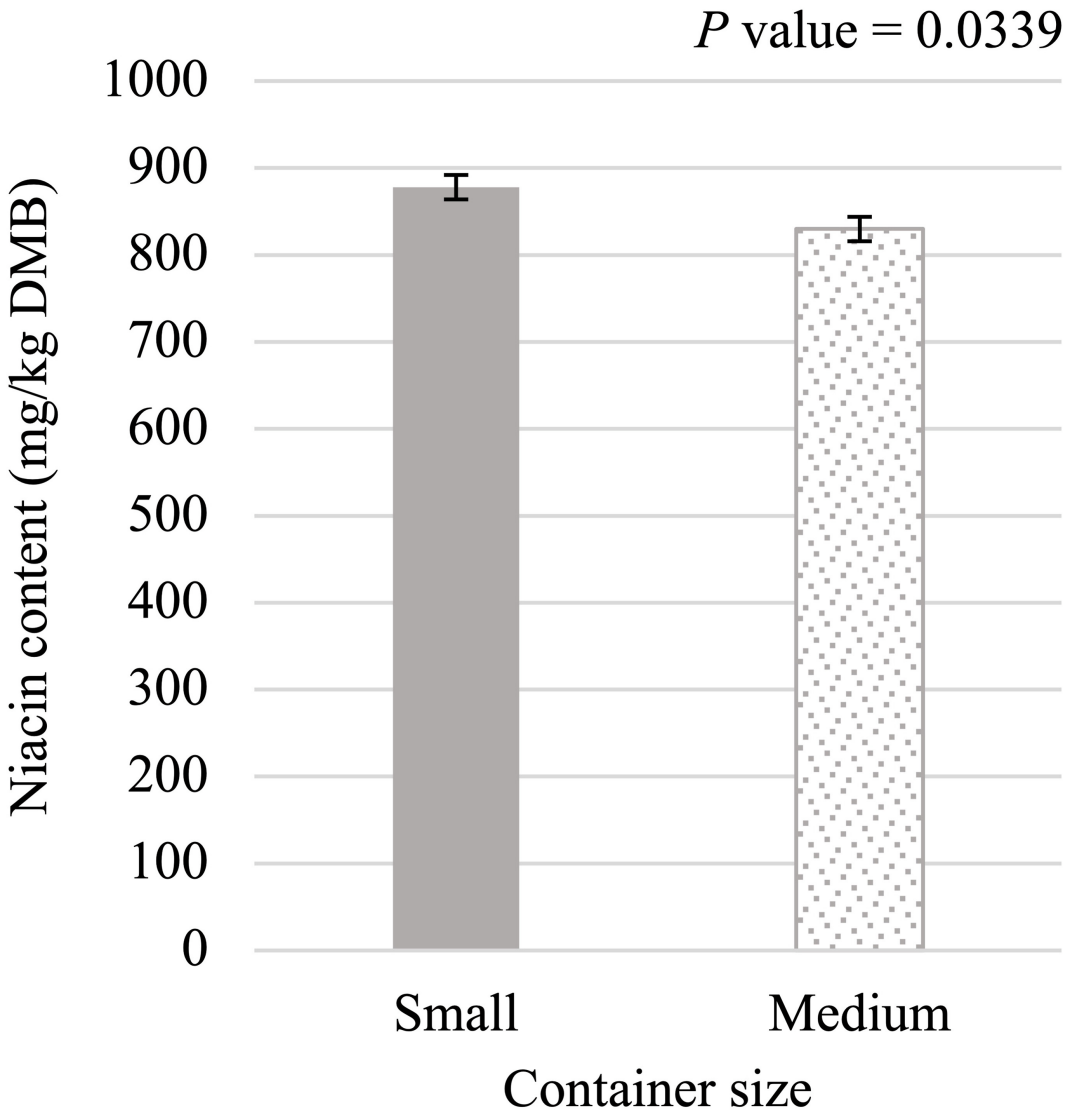
Main effect of container size on dry matter basis (DMB) niacin content (average ± pooled standard error of the mean) of canned cat food processed in containers of two different sizes and three different types.

**Figure 6:**
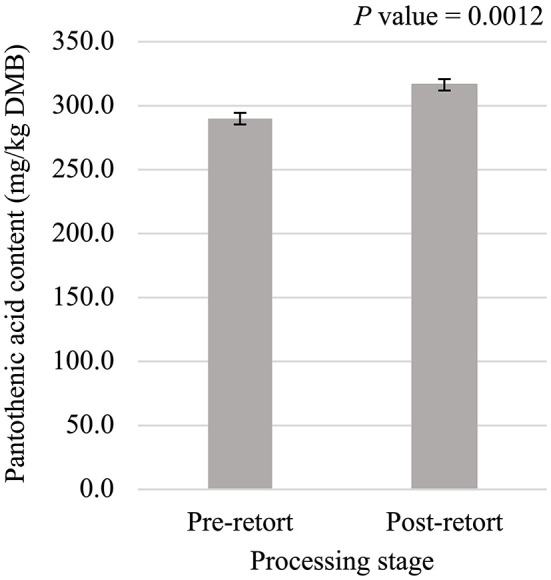
Main effect of processing stage on dry matter basis (DMB) pantothenic acid content (average ± pooled standard error of the mean) of canned cat food processed in containers of two different sizes and three different types.

**Figure 7:**
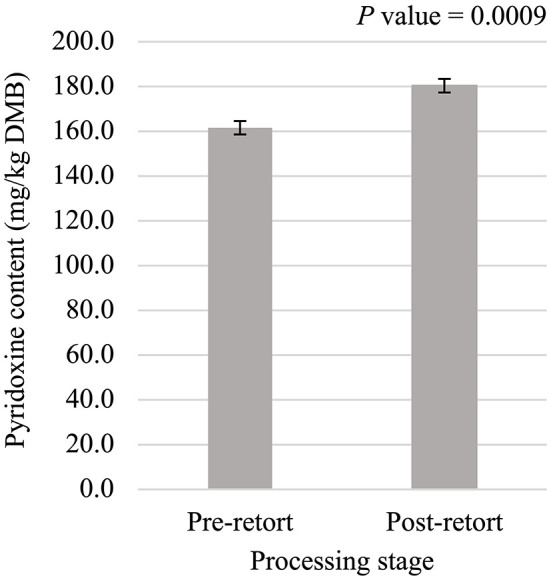
Main effect of processing stage on dry matter basis (DMB) pyridoxine content (average ± pooled standard error of the mean) of canned cat food processed in containers of two different sizes and three different types.

**Figure 8:**
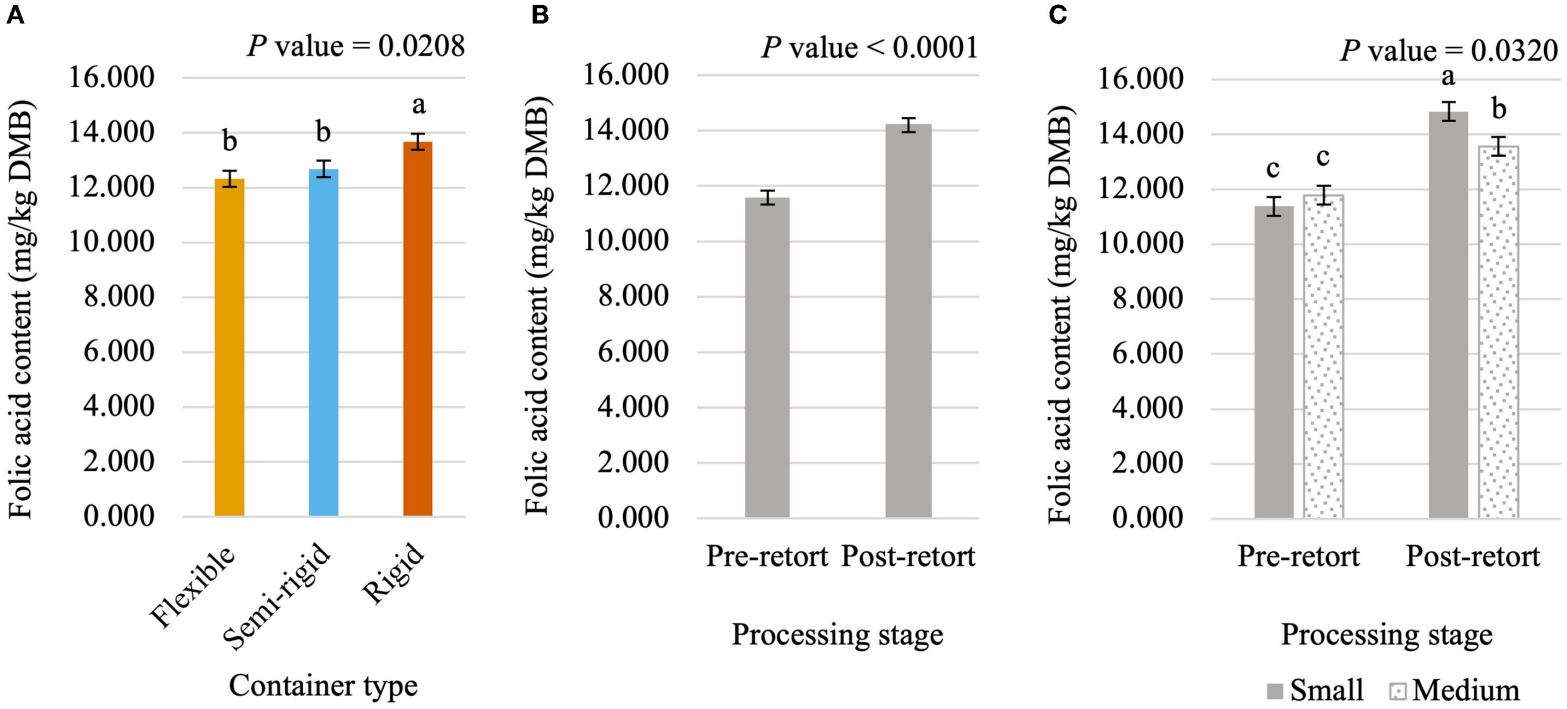
Main effects of container type **(A)** and processing stage **(B)** and the interaction of container size and processing stage **(C)** on dry matter basis (DMB) pyridoxine content (average ± pooled standard error of the mean) of canned cat food processed in containers of two different sizes and three different types. ^abc^Means within a main effect or interaction without a common superscript are different (*P* < 0.05).

Niacin content was 5.75% greater (*P* < 0.05; Figure 5) in small containers than in medium containers but was not affected (*P* > 0.05; Table 3) by container type, processing stage, or any interactions. Pantothenic acid and pyridoxine contents were higher (*P* < 0.05) in post-retort samples than in pre-retort samples by 9.1% (Figure 6) and 11.6% (Figure 7), respectively. These B-vitamins were not affected (*P* > 0.05; Table 3) by container size, container type, or any interactions. Rigid containers (13.672 mg/kg DMB) contained higher (*P* < 0.05; Figure 8A) levels of folic acid than semi-rigid and flexible containers (average 12.502 mg/kg DMB), which were not different (*P* > 0.05) from each other. Folic acid content was also greater (*P* < 0.05; Figure 8B) in post-retort samples than in pre-retort samples by 22.6%. The interaction between container size and processing stage was also significant (*P* < 0.05; Figure 8C); small containers post-retort processing (14.385 mg/kg DMB) contained the highest level of folic acid, followed by medium containers post-retort (13.565 mg/kg DMB) with pre-retort samples in small and medium containers (average 11.583 mg/kg DMB) containing the lowest level. The main effect of container size and all unmentioned interactions did not affect (*P* > 0.05; Table 3) folic acid content. Finally, biotin (average 1.528 mg/kg DMB; Supplementary Table 2) and cobalamin (average 0.4173 mg/kg DMB; Supplementary Table 2) were not affected (*P* > 0.05; Table 3) by container size, container type, processing stage, and any interactions.

## 4. Discussion

The goal of this experiment was to determine if container size and type influenced retort processing and retention of B-vitamins in canned cat food. Minimum recommended levels should be met for a pet food to be deemed “complete and balanced” in the United States (1). As such, it is important for pet food companies to ensure their products contain enough of each B-vitamin to support animal health. Other research on vitamin degradation due to retort processing has utilized rigid cans as the container type (15–17). However, flexible pouches and semi-rigid trays/tubs have become popular offerings for canned cat foods and research with human food products suggests there are differences in their processing.

### 4.1. Thermal processing metrics

Thermocouple failure was expected simply due to the large number employed in the present experiment and has been documented in other experiments (15, 18). However, the minimum number of 10 thermocouples recommended by the Institute for Thermal Processing Specialists (12) was achieved in the processing of each replicate.

Flexible and semi-rigid containers required slightly different scheduled processes than rigid containers as they are more susceptible to damage during thermal processing (19). This is evidenced by the differences in retort temperatures and internal can temperatures. The small and medium rigid cans were sturdier and could withstand faster retort come-up times than the small and medium semi-rigid and flexible containers. Other packaging-related factors of heating rates include container wall thickness, thermal conductivity of the container material, and the container's ratio of surface area to volume (4). In the present experiment, the effects of container wall thickness and thermal conductivity are included in the container type factor and the container surface area to volume ratio is captured within both factors.

Small containers were cooler at the beginning of retort processing than medium containers. The intention was to keep initial can temperatures similar across all treatments because the initial can temperature can influence the heating rate and the intensity of processing (4). In general, food products with lower initial temperatures will accumulate less lethality than food products with higher initial temperatures processed under the same scheduled process (20). The scheduled process in the present experiment was dependent on when the final containers achieved the target lethality of 8 min and may have mitigated some effects of differences in initial temperature. In future experiments, initial can temperatures could be controlled better by placing filled and sealed containers in a warm water bath during the filling and seaming processes.

The time to achieve the target lethality was affected by container size, container type, and their interaction. Thermocouples were placed in containers to measure temperature in the coldest spot, or the location that heats and cools the slowest. Therefore, the decreased time required for small containers compared to medium containers was not unexpected. Flexible containers also required less time than semi-rigid and rigid containers to achieve the target lethality. A reduction in calculated process time with the use of flexible pouches instead of rigid cans has been noted in the retort processing of shrimp in a sauce (21) and trout, pollock, and shrimp alone (22). These researchers suggested the differences in the surface area to volume ratio between the two container types contributed to their findings. As in their research, the flexible pouches in the present experiment were flatter than the semi-rigid and rigid containers, which can reduce the time required for thermal processing (23). This is echoed when the interaction between container size and container type is analyzed. Flexible containers required the shortest amount of time and the difference between medium and small flexible containers was smaller than between medium rigid or semi-rigid containers and small rigid or semi-rigid containers. Other researchers have also found a greater effect of container size on calculated process time when food was processed in rigid cans than in flexible pouches to achieve a target lethality (24). Process developers should take these differences in heat penetration into account when designing scheduled processes to minimize over-processing.

All container types and sizes were processed to average heating lethalities greater than the target of 8 min. This was not unexpected, as other researchers have also struggled to achieve target lethalities when processing canned cat foods (18, 25). Initiating the cooling cycle when the final thermocouple-containing container reached the target lethality meant all other containers would be “over-processed”, thus resulting in a higher than intended average heating lethality. However, the lack of difference in heating lethalities suggested a successful retort processing.

Internal container temperature may continue to rise during early portions of the cooling phase and contribute to accumulated lethality (26). This was observed in the present experiment, wherein the main effects of container size and container type were significant. The greater cooling cycle lethality for small containers compared to medium containers was unexpected. This finding suggested that the cooling of small containers was slower than for medium containers (20). It is important to note than only a 9% difference was observed between container sizes and this relationship was not observed for total accumulated lethality. Greater differences were observed between container types, where the greatest cooling cycle lethality was accumulated in semi-rigid containers, followed by flexible containers, and rigid containers accumulated the lowest lethality. The scheduled process for rigid containers utilized a faster retort cool-down that was not possible with the semi-rigid and flexible containers. This was required to prevent deformation of semi-rigid and flexible containers by cooling them too quickly. However, this may have led to greater cooling cycle lethalities in those container types. The fact that flexible containers accumulated less lethality during the cooling cycle than semi-rigid containers suggested a faster cooling rate similar to the differences observed in the time to achieve target lethality between container types.

Regardless of these outcomes, total accumulated lethality was only affected by container type. The grouping of semi-rigid and flexible containers accumulating more lethality than rigid containers suggested that differences in the scheduled process for these container types, particularly during the cooling cycle, played a role in the overall processing intensity. Nevertheless, total accumulated lethality for all six treatments was similar to the reported typical lethality of 12–14 min for commercial canned cat foods (25).

### 4.2. B-vitamin content of pre- and post-retort samples

The addition of vitamin premix was necessary to meet the intended thiamin level target prior to retort processing as well as the minimum recommended level for cats in adult maintenance or growth and reproduction [5.6 mg/kg DMB (1)]. This agrees with previous research that found low levels of thiamin in ingredients used to manufacture chicken-based canned cat foods (15). Levels of other B-vitamins were also low in pre-retort samples without vitamin premix in the present experiment. Undetectable levels of riboflavin, niacin, and pyridoxine indicated that other ingredients present in the canned cat food did not provide meaningful contributions. As such, supplementation of these vitamins through the inclusion of a vitamin premix is necessary to meet the minimum recommended levels of cats [4.0 mg/kg DMB; 60 mg/kg DMB, and 4.0 mg/kg DMB, respectively (1)]. It is likely that the mechanically separated chicken, brown rice, and dried egg product provided minimal levels of the other B-vitamins. All three ingredients have been documented with measurable levels of pantothenic acid and folic acid while only dried whole egg contained measurable levels of cobalamin (27). Regardless, the minimum recommended levels of pantothenic acid, biotin, and cobalamin for adult maintenance and growth and reproduction for cats [5.75 mg/kg DMB, 0.07 mg/kg DMB, and 0.020 mg/kg DMB, respectively (1)] were met without the addition of vitamin premix prior to retort processing. The level of folic acid did not meet the recommended level [0.8 mg/kg DMB (1)] without the vitamin premix, indicating that inclusion of a vitamin premix is also necessary for this vitamin.

The main effect of container size was significant for thiamin, riboflavin, and niacin contents and the main effect of container type was significant for folic acid content. This indicated low levels of variability in the addition of the vitamin premix to the formula and/or in the vitamin analysis. Analysis of these main effects did not differentiate between pre-retort and post-retort samples; these main effects do not suggest improved vitamin retention with a specific container size or type.

Thiamin degradation due to thermal processing is well-documented in canned foods. Experimentally, thiamin degradation in canned cat food ranged from 45.8 to 82.9% when different thiamin sources were included in the diet (15). On the other hand, industry recommendations describe thiamin degradation due to processing as high as 90% (1). These reports are much higher than the average 30.4% thiamin loss observed in the present experiment. None of the interactions between processing stage and container size and/or container type were significant in the present experiment. This does not agree with research that documented greater thiamin retention in rainbow trout, pollock, and shrimp processed in pouches instead of cans (22). However, statistical analysis was not used to make this conclusion. Other researchers identified a trend for lower thiamin retention when cat food was processed in 156 g cans vs. 349 g cans (16). That research group's experimental design included processing time as a factor, which may have led to smaller cans accumulating greater lethality during the heating cycle. If this were the case, it is reasonable to assume the cooling cycle exacerbated this difference and confounds the data. Heating lethality was not different among the treatments in the present experiment and total lethality for semi-rigid and flexible containers by 2.13 min. This suggests there may be a range of total lethalities before differences in thiamin degradation are observed, as the interaction between container type and processing stage was not significant. Others have found nearly twice the thiamin degradation when a canned cat food containing thiamin mononitrate was processed to a total lethality around 80 min (15). Future research should process one canned cat food formulation to increasing total lethalities and measure thiamin before and after processing to determine how much overprocessing is acceptable.

This is the first experiment to document decreased riboflavin content in canned cat food due to thermal processing. The 81.7% retention observed was not unexpected. Retention of riboflavin in beef or veal home-canned in different sizes of cans and jars ranged from 83 to 112% and were not considered significant (28). Cut potatoes and cow peas also retained 83.7 and 85.2%, riboflavin, respectively, after the retorting process (29). However, riboflavin retention was lower for retorted tuna [49.8%; (30)] and for immature seeds of varieties of the common bean [44.3–59.1%; (31)]. It is challenging to compare riboflavin retention across experiments with different processing conditions without a common measure of processing intensity, such as total lethality. Future research could investigate the effect of total lethalities on riboflavin retention in a canned cat food. However, the higher riboflavin retention compared to thiamin retention in the present experiment suggested that thiamin may be more important when optimizing formulation and the scheduled process.

The B-vitamins niacin, biotin, and cobalamin were not affected by retort processing in this experiment. Niacin is considered stable during food processing, but retention is affected when retorting temperature and time are altered (32, 33). These factors were kept as consistent as possible in the present experiment but could not be exact due to the constraints of the flexible and semi-rigid container types. It appeared the slightly higher total lethality accumulated by semi-rigid and flexible containers was not great enough to influence niacin retention. Very little is published about the retention of cobalamin during retort processing. Preliminary findings saw no difference in cobalamin content when canned pet food was processed for 45, 60, or 90 min in a retort (17). This indicated that cobalamin is not heat-labile and agreed with the findings in the present experiment. Even less is published regarding the effect of retort processing on biotin content; this may be due to minimal instances when biotin supplementation in pet food is necessary (1). It appeared that niacin, cobalamin, and biotin were stable during the retort process when minimum heating lethality values were targeted in the present experiment. This may not be the case if the canned cat foods were processed to higher lethality values and could be addressed in future experiments.

Post-retort samples were higher in pantothenic acid, pyridoxine, and folic acid compared to pre-retort samples. This finding is counter-intuitive and was more than likely influenced by sampling variation and analytical variation, leading to the conclusion that pantothenic acid, pyridoxine, and folic acid are minimally affected by retort processing. Reports of pantothenic losses due to retort processing range from 19.7% [water-blanched spinach (34)] to around 30.0% [home-canned beef and veal (28)]. However, other researchers found that pantothenic acid is not heat-labile and suggested the vitamin may be more protected in food systems than in model systems (35). Similarly, preliminary research with canned cat food identified minimal differences in pantothenic acid content when retort processing time was increased (17). There is greater consensus for pyridoxine and folic acid. Losses of pyridoxine in strained lima beans, stained beef, and tomato juice were 10% or lower and were similar between conventional retorting and high temperature-short time aseptic processing (36). This led researchers to conclude that pyridoxine is minimally impacted by heat processing. Pyridoxine and folic acid were also stable during retort processing of pinto beans (37) soybeans (38) when variability and leaching of vitamins into cooking water were considered. These vitamins are important in supporting pet cat health, but degradation is not a major concern when canned cat foods are processed to minimum intensities.

## 5. Conclusions

Processing of flexible and semi-rigid containers required different retort processing settings than rigid containers. This was evident in the greater accumulation of lethality during the cooling cycle for these containers and was likely due to the slower cooling of containers to prevent deformation. Flexible containers required less time to achieve target lethality than semi-rigid and rigid containers and small containers required less time than medium containers. Pet food processors should take these findings into consideration when designing the scheduled processes for flexible and semi-rigid containers and create processes that balance food product changes due to over-processing and the risk of not meeting regulations due to under-processing.

Vitamin premix supplementation was necessary to meet minimum recommended levels of thiamin, riboflavin, niacin, pyridoxine, and folic acid. Retort processing decreased levels of thiamin and riboflavin, but container size and container type did not influence degradation differently even though containers were processed to slightly different total lethalities. The other B-vitamins (niacin, pantothenic acid, pyridoxine, folic acid, biotin, and cobalamin) were stable during retort processing. However, it is important to remember that foods were processed to low lethalities and these vitamins could be more sensitive if processing was more intensive. Future research in this area should process the same formula in the same container to increasing lethalities to evaluate this. The same experiment could also identify the accumulated lethality a canned cat food must be processed to for a difference in thiamin and/or riboflavin degradation to be observed. Based on the data in this experiment, pet food formulators should formulate canned cat foods with higher than necessary levels of thiamin and riboflavin to account for processing losses. They should also consider over-formulating the other B-vitamins if the processing facility cooks to higher lethalities than done in this experiment.

## Data availability statement

The raw data supporting the conclusions of this article will be made available by the authors, without undue reservation.

## Author contributions

CA designed the experiment. LM performed the experiment. AD performed the statistical analysis and wrote the manuscript. All authors revised and provided intellectual input on this manuscript. All authors contributed to the article and approved the submitted version.
