# Supplementary material for: Effects of container type and size on thermal processing characteristics and B-vitamin retention of canned cat food
Source: Front Vet Sci. 2023 May 11;10:1175819. doi: 10.3389/fvets.2023.1175819 (PMC10213778; doi:10.3389/fvets.2023.1175819)
Supplement: Supplementary file 1 [file Table_1.DOCX]

Supplementary Material

Effects of container type and size on thermal processing characteristics and B-vitamin retention of canned cat food

Amanda N. Dainton, Lydia M. Molnar, Charles Gregory Aldrich^*^

*** Correspondence:** Corresponding Author: aldrich4@ksu.edu

**Supplementary Table 1: Quality control measurements (average ± standard deviation) of pre-retort and post-retort canned pet food processed in two different container sizes and three different container types.**

|  | Small | | | Medium | | |
| --- | --- | --- | --- | --- | --- | --- |
| Measurement | Flexible | Semi-rigid | Rigid | Flexible | Semi-rigid | Rigid |
|  | --------------------------------------- Container characteristics --------------------------------------- | | | | | |
| Pre-retort burst test, kPa | 150.3 ± 6.45 | 75.0 ± 3.08 | N/A | 95.5 ± 6.92 | 50.2 ± 4.09 | N/A |
| Post-retort burst test, kPa | 146.0 ± 0.05 | 70.6 ± 0.04 | N/A | 92.5 ± 5.27 | 51.0 ± 3.36 | N/A |
| Pre-retort vacuum, kPa | N/A | N/A | -18.6 ± 7.18 | N/A | N/A | -20.3 ± 0.00 |
|  | ------------------------------------------ Food characteristics ------------------------------------------ | | | | | |
| Pre-retort moisture, % | 78.2 ± 0.35 | 78.6 ± 0.07 | 78.4 ± 0.32 | 78.1 ± 0.04 | 78.4 ± 0.11 | 78.4 ± 0.07 |
| Post-retort moisture, % | 78.0 ± 0.57 | 78.1 ± 0.14 | 78.1 ± 0.49 | 77.6 ± 0.04 | 78.4 ± 0.07 | 78.3 ± 0.11 |
| Post-retort crude protein, % DMB^1^ | 31.8 ± 0.31 | 32.3 ± 0.08 | 32.7 ± 0.28 | 31.2 ± 1.12 | 32.1 ± 0.04 | 31.4 ± 0.58 |
| Post-retort crude fat, % DMB^1^ | 47.4 ± 0.07 | 47.7 ± 0.01 | 47.2 ± 0.58 | 48.3 ± 0.87 | 48.0 ± 0.17 | 47.6 ± 0.72 |
| Post-retort ash, % DMB^1^ | 9.46 ± 0.014 | 9.47 ± 0.165 | 9.68 ± 0.090 | 9.32 ± 0.268 | 9.58 ± 0.067 | 9.26 ± 0.069 |
| Pre-retort pH | 5.94 ± 0.004 | 6.01 ± 0.057 | 5.90 ± 0.025 | 6.07 ± 0.092 | 6.03 ± 0.021 | 6.02 ± 0.011 |
| Post-retort pH | 6.50 ± 0.004 | 6.49 ± 0.018 | 6.47 ± 0.000 | 6.41 ± 0.134 | 6.47 ± 0.000 | 6.46 ± 0.007 |
| ^1^ DMB = dry matter basis | | | | | | |

**Supplementary Table 2: Dry matter basis B-vitamin content (average ± standard deviation) of canned cat food samples from two container sizes, three container types, and two processing stages.**

| Processing stage | Small | | | Medium | | |
| --- | --- | --- | --- | --- | --- | --- |
|  | Flexible | Semi-rigid | Rigid | Flexible | Semi-rigid | Rigid |
|  | ------------------------------------------------------- Thiamin, mg/kg ------------------------------------------------------- | | | | | |
| Pre-retort | 3541.2 ± 0.67 | 3427.8 ± 9.65 | 3526.9 ± 31.48 | 3271.1 ± 399.48 | 3423.0 ± 7.00 | 3521.0 ± 137.24 |
| Post-retort | 2635.7 ± 30.19 | 2392.6 ± 36.21 | 2452.7 ± 58.66 | 2336.3 ± 109.56 | 2330.1 ± 77.31 | 2266.3 ± 72.10 |
|  | ------------------------------------------------------- Riboflavin, mg/kg ------------------------------------------------------- | | | | | |
| Pre-retort | 110.9 ± 2.93 | 113.3 ± 7.88 | 106.0 ± 5.05 | 98.5 ± 8.63 | 108.1 ± 5.54 | 101.2 ± 3.47 |
| Post-retort | 88.6 ± 0.88 | 87.1 ± 1.22 | 88.7 ± 8.07 | 85.6 ± 5.89 | 86.9 ± 1.60 | 84.7 ± 1.01 |
|  | ------------------------------------------------------- Niacin, mg/kg ------------------------------------------------------- | | | | | |
| Pre-retort | 854 ± 48.5 | 897 ± 120.2 | 934 ± 30.3 | 795 ± 55.8 | 900 ± 31.8 | 853 ± 7.0 |
| Post-retort | 876 ± 25.0 | 847 ± 14.9 | 859 ± 2.6 | 824 ± 22.7 | 800 ± 71.2 | 808 ± 14.0 |
|  | -------------------------------------------------- Pantothenic acid, mg/kg -------------------------------------------------- | | | | | |
| Pre-retort | 299.3 ± 2.90 | 287.0 ± 15.78 | 302.0 ± 0.52 | 289.4 ± 3.08 | 305.9 ± 24.06 | 255.6 ± 38.03 |
| Post-retort | 342.9 ± 6.87 | 313.7 ± 2.67 | 314.5 ± 6.78 | 303.9 ± 4.90 | 314.6 ± 18.66 | 308.6 ± 10.23 |
|  | ------------------------------------------------------- Pyridoxine, mg/kg ------------------------------------------------------- | | | | | |
| Pre-retort | 163.4 ± 4.48 | 168.5 ± 8.35 | 165.3 ± 3.13 | 147.1 ± 9.59 | 167.5 ± 8.03 | 158.0 ± 2.48 |
| Post-retort | 181.9 ± 11.37 | 185.9 ± 2.17 | 170.0 ± 29.35 | 170.3 ± 6.68 | 192.7 ± 2.80 | 181.5 ± 1.40 |
|  | ------------------------------------------------------- Biotin, mg/kg ------------------------------------------------------- | | | | | |
| Pre-retort | 1.457 ± 0.1285 | 1.518 ± 0.0545 | 1.617 ± 0.0990 | 1.460 ± 0.0733 | 1.567 ± 0.0251 | 1.568 ± 0.0308 |
| Post-retort | 1.652 ± 0.1258 | 1.669 ± 0.0140 | 1.083 ± 0.6476 | 1.580 ± 0.1194 | 1.616 ± 0.1254 | 1.555 ± 0.0337 |
|  | ------------------------------------------------------- Folic acid, mg/kg ------------------------------------------------------- | | | | | |
| Pre-retort | 10.978 ± 0.7932 | 10.862 ± 0.0301 | 12.297 ± 0.5384 | 10.286 ± 0.5649 | 12.095 ± 0.9893 | 12.981 ± 1.0875 |
| Post-retort | 14.758 ± 0.2948 | 14.526 ± 1.7728 | 15.219 ± 0.7861 | 13.273 ± 0.4215 | 13.234 ± 0.5331 | 14.189 ± 0.9503 |
|  | ------------------------------------------------------- Cobalamin, mg/kg ------------------------------------------------------- | | | | | |
| Pre-retort | 0.4478 ± 0.00667 | 0.3998 ± 0.02275 | 0.4381 ± 0.01088 | 0.4071 ± 0.03194 | 0.4225 ± 0.06872 | 0.4288 ± 0.04139 |
| Post-retort | 0.4376 ± 0.03623 | 0.3835 ± 0.01173 | 0.4218 ± 0.03149 | 0.3832 ± 0.05723 | 0.3979 ± 0.02973 | 0.4399 ± 0.01422 |
